# The *Arabidopsis* Calcium-Dependent Protein Kinases (CDPKs) and Their Roles in Plant Growth Regulation and Abiotic Stress Responses

**DOI:** 10.3390/ijms19071900

**Published:** 2018-06-28

**Authors:** Sujuan Shi, Shugui Li, Muhammad Asim, Jingjing Mao, Dizhi Xu, Zia Ullah, Guanshan Liu, Qian Wang, Haobao Liu

**Affiliations:** 1 Tobacco Research Institute, Chinese Academy of Agricultural Sciences, Qingdao 266101, China; shisujuan2014@163.com (S.S.); lishugui88@163.com (S.L.); asim.ktk91@aup.edu.pk (M.A.); maojingjing40@163.com (J.M.); dizhixu@foxmail.com (D.X.); zia_nust@yahoo.com (Z.U.); liuguanshan@caas.cn (G.L.); 2 College of Agriculture, Qingdao Agricultural University, Qingdao 266109, China

**Keywords:** calcium, CDPK, protein structure, growth, abiotic stress, *Arabidopsis*

## Abstract

As a ubiquitous secondary messenger in plant signaling systems, calcium ions (Ca^2+^) play essential roles in plant growth and development. Within the cellular signaling network, the accurate decoding of diverse Ca^2+^ signal is a fundamental molecular event. Calcium-dependent protein kinases (CDPKs), identified commonly in plants, are a kind of vital regulatory protein deciphering calcium signals triggered by various developmental and environmental stimuli. This review chiefly introduces Ca^2+^ distribution in plant cells, the classification of *Arabidopsis thaliana* CDPKs (AtCDPKs), the identification of the Ca^2+^-AtCDPK signal transduction mechanism and AtCDPKs’ functions involved in plant growth regulation and abiotic stress responses. The review presents a comprehensive overview of AtCDPKs and may contribute to the research of CDPKs in other plants.

## 1. Introduction

Plants respond to environmental fluctuations and developmental cues by generating intricate signal transduction networks, which are composed of multiple protein or non-protein elements. The former includes disparate receptors, enzymes and transcription factors, while the latter mainly refers to some secondary messengers, such as calcium (Ca^2+^), cyclic nucleotides, active oxygen species, lipids and hydrogen ions. Among them, Ca^2+^ is the most important secondary messenger [1,2,3]. Besides the above, Ca^2+^ is also required for maintaining the stability of the cell wall and membranes, regulating physiological processes, including the stomatal guard cell movement, root hair elongation and pollen tube growth, and functions as an essential plant nutrient [4,5]. When plants undergo various environmental and developmental stimuli, specific spatiotemporal calcium signals are elicited in the form of transient changes in Ca^2+^ concentration in cells. The stimuli, triggered by temperature, light, salt, osmotic stress and other kinds of external factors, can generate diverse Ca^2+^ changes, and these changes can be recognized and sensed by particular calcium sensors/receptors to induce further output transcriptional and metabolic responses [6].

Most Ca^2+^ sensors recognize Ca^2+^ signals via the elongation factor hand (EF-hand) motif, a characteristic and conserved helix–loop–helix structure that binds one Ca^2+^ ion. EF-hands have a tendency to occur in pairs as a discrete domain, so that most Ca^2+^ sensor family members possess two, four or six EF-hands [6,7]. The pairing in certain conditions shows a positive co-operativity, thereby minimizing the Ca^2+^ signal required to reach protein saturation. Ca^2+^ binding to the Ca^2+^ sensor elicits a structural change that promotes the interaction between the sensor and its target proteins or changes the enzyme activity of the Ca^2+^ sensor. There are a large set of EF-hand-containing proteins in plants, and Ca^2+^ sensors are one general class that translates the chemical signals into diverse biochemical responses [6,7].

So far, several main Ca^2+^ sensor families in plants have been discovered: Calmodulins (CaMs), Calmodulin-like proteins (CMLs), Calcineurin B-like proteins (CBLs) and Ca^2+^-dependent protein kinases (CDPKs) [8,9]. CaM is highly conserved in all eukaryotes, whereas CMLs, CBLs and CDPKs are only identified in plants and some protists [10,11]. Among these families, CaMs, CMLs and CBLs are small-molecule proteins only harboring a Ca^2+^ sensing domain; they therefore work as sensor relays by binding to the downstream effectors in a Ca^2+^-concentration dependent manner [12]. Significantly different from those above, CDPKs contain a serine/threonine protein kinase catalytic domain as an effector domain as well as possessing a Ca^2+^ sensing domain. Therefore, CDPKs are able to activate and regulate the target proteins directly when they sense the Ca^2+^ signals. In short, CDPKs function as direct “sensor responders” to decode the Ca^2+^ signals [13].

To date, CDPKs have commonly presented in plants, protists, oomycetes and green algae, but not in animals and fungi [3]. CDPKs constitute a large multi-gene kinase family in various plant species and are reported to play important roles in plant growth, development and stress responses. There are 34 CDPKs which have been identified in *Arabidopsis thaliana* (*AtCDPKs*), 31 in *Oryza sativa*, 35 in *Zea mays* and 20 in *Populus trichocarpa*, and some crucial CDPKs have been deeply researched [2]. Although considerable work has been done to elucidate CDPKs’ decoding mechanism and Ca^2+^-CDPKs signaling networks, the understanding of this field is fragmentary. In this review, we mainly introduce the basic introduction of the Ca^2+^ signaling pathway and AtCDPKs’ functions involved with plant growth regulation and abiotic stress responses.

## 2. The Storage and Distribution of Calcium in Plant Organelles

Ca^2+^ enters plant cells via membrane-localized Ca^2+^-permeable channels. To avoid it forming insoluble precipitates with phosphate, Ca^2+^ does not diffuse freely in the cell, but is mainly stored at much higher concentrations (~mM) in various plant organelles, including vacuole, nucleus, endoplasmic reticulum (ER), chloroplast, mitochondria and apoplast [14,15]. The cytoplasmic Ca^2+^ concentration is kept by Ca^2+^-ATPases and H^+^/Ca^2+^-antiporters at a sufficiently low level (~100 nM) during a resting state, and it can be sharply raised to a millimol via the Ca^2+^ influx regulated by these membrane-localized cation channels in the surrounding organelles [13,14,15]. Different organelles of plant cells might have various Ca^2+^ concentrations [14,16]. The nature of the diverse stimuli the plants perceive determines the shape of the cytosolic Ca^2+^ signals and finally forms the stimulus-specific pattern of Ca^2+^ pulses, which are termed as Ca^2+^ signatures. The physiobiological basis of Ca^2+^ signatures is the activities and expression of different Ca^2+^ pumps and channels as well as other uncharacterized factors and interactions. All of these determine the peak amplitudes, duration and frequency of Ca^2+^ transients cooperatively [4,13,17]. The Ca^2+^ changes are then recognized by Ca^2+^ sensors and induce arrays of responses in plants. Therefore, the instantaneous fluctuations of Ca^2+^ distribution and gradients trigger the complicated cellular networks to react to the changing environmental and developmental conditions [4].

It is very difficult to measure the ionic concentration (or activities) of elements in a sample of plant tissue accurately. Generally speaking, the free ion concentration is usually less than the total elemental content. The calcium ion is always complex or bound irreversibly to other agents. The main techniques available to measure the elemental or ionic content of plant tissues with a resolution from the whole plant to the sub-cellular level are covered by the review written by Con and Gilliham [15]. The vacuole is the major storage compartment of calcium in plant cells. Most of the calcium is often tightly bound to chelating agents or to some special Ca^2+^-binding proteins. The free vacuolar Ca^2+^ concentration always ranges from 0.2 to 1~5 mM, while the total calcium concentration can reach a maximum of 80 mM, observed by using X-ray microanalysis (XRMA) [14,15]. The total calcium concentration in the chloroplast is about 15 mM or higher, and it is up-regulated by light [14]. Most of the calcium is bound to thylakoid membranes or stromal proteins to avoid the photosynthesis inhibition of higher levels of calcium [18]. The apoplast is another important place of storage of Ca^2+^ ions. Ca^2+^ movement here depends on the water transpiration stream [19]. Notably, the vacuole and apoplast both possess a high potential for Ca^2+^ unloading, and the sequestration of higher calcium amounts functions not only in signaling but also in the basic ion homeostasis of the plant. The free Ca^2+^ concentration in the cytoplasm and nucleus is basically ~100 nM [14]. As a result of the lack of molecular identification of plant ER-located Ca^2+^ channels, little is known about ER Ca^2+^ storage in plants. It is considered that calreticulin is the major Ca^2+^-storage protein in ER [14,15]. A brief overview of Ca^2+^ concentration in organelle stores in plants is summarized below (Table 1).

## 3. Classification and Localization of *Arabidopsis* CDPKs

**Figure 1:**
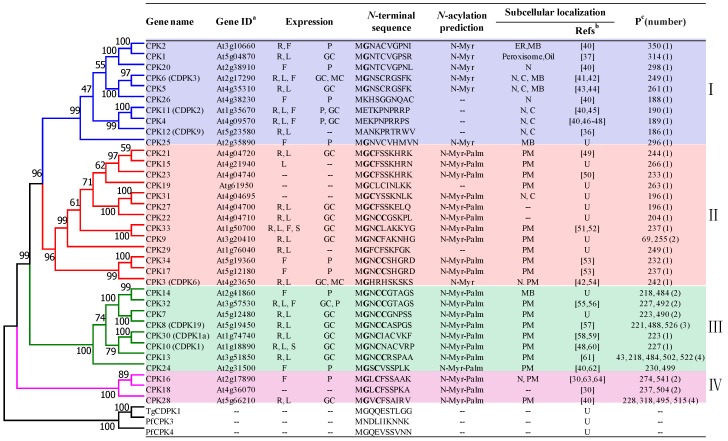
Phylogenetic relationships and characteristics of selected calcium-dependent protein kinases (CDPKs) (modified from [25]). The full-length amino acid sequences of CDPKs from *A. thaliana*, *P. falciparum*, and *T. gondii* were analyzed by MEGA7.0 and UniProt (https://www.uniprot.org/). *Arabidopsis thaliana* CDPKs (AtCDPKs) are divided into four major subgroups (I–IV). R, root; L, leaf; F, flower; S, silique; GC, guard cell; P, pollen tube cell; MC, mesophyll cell; N, nucleus; C, cytoplasm; PM, plasma membrane; MB, membrane; ER, endoplasmic reticulum; --, not determined. **G**, the *N*-myristoylation site. **C**, the palmitoylation site. U, UniProt Knowledgebase. ^a^ Systematic designation given to a gene according to TAIR (www.arabidopsis.org) and UniProt (www.uniprot.org). ^b^, References related to subcellular localization of CPKs: [30,36,37,40,41,42,43,44,45,46,47,48,49,50,51,52,53,54,55,56,57,58,59,60,61,62,63,64]. ^c^, the position of phosphoserine site.

There are 34 CDPK proteins (also abbreviated as CPK) identified in *A. thaliana* [9]. A phylogenetic tree is constructed based on the amino acid sequences of all CDPKs from *A. thaliana* (AtCDPKs), *Plasmodium falciparum* CPK3 (PfCDPK3) and CPK4 (PfCDPK4), and *Toxoplasma gondii* CDPK1 (TgCDPK1). The phylogenetic analysis demonstrated that all CDPKs in *A. thaliana* can be divided into four subgroups (Figure 1) [25]. Compared to the other three subgroups, subgroup IV, comprising CPK16, CPK18 and CPK28, is the closest gene branch to that of the protists.

Protein autophosphorylation is commonly observed on serine and threonine residues of most CDPK members. Extensive studies have demonstrated that this autophosphorylation always occurs in a Ca^2+^-dependent manner and prompts kinase activity [26,27]. A statistical analysis of the phosphorylation residues in AtCDPKs demonstrated that all members except CPK9 in subgroup I and II have only one phosphoserine position, while this is not fixed in those from subgroup III and IV, where the number of positions ranges from one to four. How the serine residue affects Ca^2+^ binding and kinase activity is unclear [28,29].

CDPKs are distributed widely in plants and have a ubiquitous expression in different tissues, including roots, leaves, flowers, siliques, etc. Some CDPKs have a widespread distribution in most tissues while others are expressed specifically [30,31,32]. At the cellular level, CDPKs present abundantly in meristem, xylem, pollen, guard cells and embryonic cells (Figure 1) [25]. CDPKs have a diverse subcellular localization, including the cytosol, nucleus, plasma membrane (PM), ER, tonoplast, mitochondria, chloroplasts, oil bodies and peroxisomes (Figure 1) [2,25]. Widespread distribution of CDPKs in plants suggests that they have great potential to activate expansive substrates and play significant roles in numerous signal transduction pathways [2,25,33].

Most AtCDPKs are found to be associated with various membranes in plant cells. It is considered that the myristoylation plays a role in CDPKs’ membrane targeting [34]. Researchers found that, although the *N*-terminal leader sequences of AtCDPKs vary in amino acid composition and length, most of them harbor a putative *N*-terminal myristoylation motif, and 30 out of 34 AtCDPK members have a conserved *N*-terminal glycine residue at the second position, which might be myristoylated (covalently attached by a C 14:0 fatty acid) under certain circumstances (Figure 1) [34]. In many cases, it has been verified that *N*-terminal myristoylation prompts protein membrane targeting and protein–protein interaction [34]. Research was then conducted to unveil the relationship between *N*-terminal glycine myristoylation and the membrane association. It was shown that the mutation of *N*-glycine residue abolishes the *N*-myristoylation and thus the protein’s membrane association [35]. In accordance with the result, four AtCDPK proteins (CPK4, CPK11, CPK12 and CPK26) without an *N*-myristoylation site have been observed to be located in the cytosol and nucleus [36,37]. However, a lack of sufficient experimental evidence hampers the elucidation of the relationship between myristoylation and membrane targeting. Among the 30 AtCDPKs with conserved *N*-glycine residue, only CPK2 has been identified as being myristoylated at the *N*-glycine residue experimentally [38]. It seems that other uncharacterized mechanisms or factors might also contribute to the membrane-anchoring processes. Supportively, CPK5 and CPK6 without myristoylation were found to associate with the membrane partially [39]. Palmitoylation (the addition of palmitate, a C 16:0 fatty acid), a second lipid-modification, has been determined to play an assisting role for CDPK membrane association [34]. For example, CPK34 and CPK2 were observed to localize on the PM, and the point mutation of the myristoylation or palmitoylation sites abolishes the PM localization and results in a cytoplasmic distribution [40].

## 4. The Typical Structure of AtCDPKs and Their Ca^2+^ Decoding Mechanism

**Figure 2:**
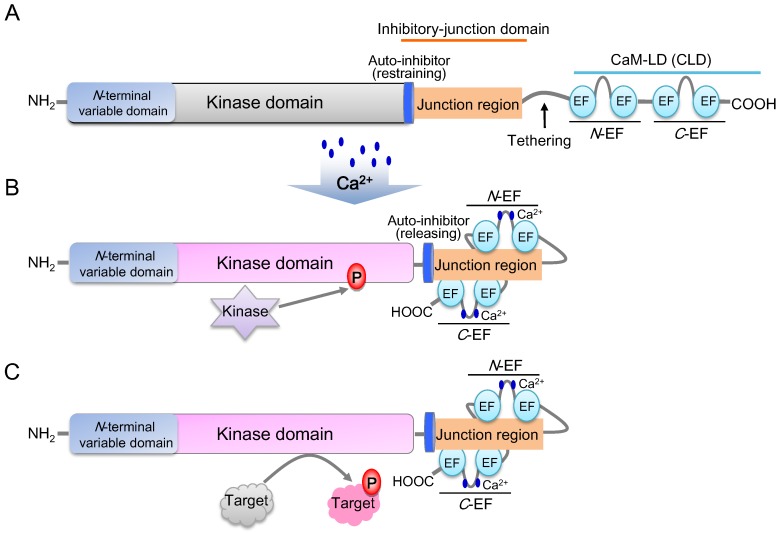
Diagram of CDPK structure and the Ca^2+^-CDPK decoding Mechanism (modified from [68]). (**A**) Inactive state of the CDPK protein. The auto-inhibitor restrains the kinase activity, while EF-hand motifs do not bind to the junction region. (**B**) Active state of the CDPK protein. After Ca^2+^ uploading into elongation factor (EF) hands, *N*-EF and *C*-EF hands combine with different sides of junction region, then the kinase domain is released and phosphorylated simultaneously by an upstream kinase. (**C**) Phosphorylation of target proteins.

The structures of all AtCDPK protein members are predicted to be highly conserved. Generally, a typical CDPK harbors four domains, including an *N*-terminal variable domain, a serine/threonine protein kinase domain, an inhibitory-junction domain and a calmodulin-like domain (CaM-LD) (Figure 2) [34]. The inhibitory-junction domain encompasses a pseudosubstrate auto-inhibitor (20~30 amino acids) and a junction region at its *C*-terminus, while the CaM-LD domain contains four EF-hands (designated as EF1-EF4) which form the *N*- and *C*-terminal EF-hand pair (*N*-EF/*C*-EF lobe) [1,65]. The covalent tethering of the CaM-LD to its regulatory-junction region in CDPKs is a unique feature within the CaM superfamily [66].

Under an inactive state, the auto-inhibitor binds to the adjacent kinase domain and restrains its activity (Figure 2A). Upon activation by Ca^2+^, a Ca^2+^ ion loads into the EF-lobes and triggers the conformational shift of CaM-LD. Meanwhile, the EF-lobes adhere to the junction region to release the auto-inhibitor. The kinase domain is then exposed and phosphorylated by an uncharacterized cellular kinase (Figure 2B) [1,67,68]. The activated CDPK is then able to recognize and phosphorylate its target proteins (Figure 2C). Auto-phosphorylation of CDPKs is found to enhance the enzyme activity [34], and it was reported that a *C*-EF lobe has a significantly higher Ca^2+^ affinity than an *N*-EF lobe, while Ca^2+^ affinity to *C*-EF hands may enhance the Ca^2+^ affinity of *N*-EF hands in AtCDPKs [1,66,67].

**Figure 3:**
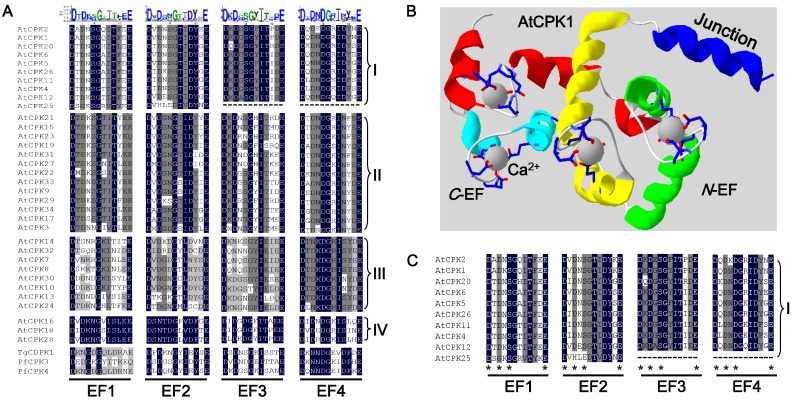
Amino acid sequence analysis of Ca^2+^-binding sites (the loop segment of the EF-hand) in AtCDPKs. (**A**) The amino acid sequences of four EF-hands in AtCDPKs, TgCDPK1, PfCDPK3 and PfCDPK4. The black line in subgroup I represents EF-hand deficiency of CPK25. (**B**) The monomeric structure of CPK1 J-CaM-LD region. The crystal structure of J-CaM-LD (PBD entry No. 2AAO) downloaded from PDB database is visualized by Swiss-PdbViewer (https://spdbv.vital-it.ch/). The Junction region that contains 31 amino acids is showed by a blue ribbon, while the helices of four EF-hands (EF1~EF4) are displayed by yellow, green, red and brilliant blue ribbons, respectively. The binding loops are displayed by grey bars. Each calcium ion binding to the loop is shown by a gray ball and the chemical bonds accountable for Ca^2+^-loop interaction are indicated by sticks. (**C**) Amino acids of four EF loops in charge of Ca^2+^-binding in CPK1 (or subgroup I) are indicated by asterisks.

Each EF-hand motif consists of a helix–loop–helix structure, and the loop segment (also termed as the Ca^2+^-binding site) composed of 12 amino acids confers the Ca^2+^-binding activity. The negatively charged amino acids in the loop are in charge of the binding [1]. A sequence analysis of Ca^2+^-binding loops in AtCDPKs demonstrates that peptides containing the 12 amino acids in each subgroup are under a high conservation (Figure 3A). To date, none of the intact plant CDPKs were crystallized; only the crystal structure of the junction region-CaM-LD (J-CaM-LD) of CPK1 is obtained (PBD entry No. 2AAO) (Figure 3B) [66]. The structure reveals a symmetric dimmer of CPK1 J-CaM-LD with a domain-swap interaction. In this model, the inhibitory-junction domain of one protomer interacts with the *C*-EF lobe of the partner protomer. The activated monomeric J-CaM-LD was obtained in solution; thus, some aspects of the intra-molecular recognition of the two domains were explained. It was noted that there is a strong interaction between the *N*- and *C*-lobes of the CaM-LD, while only the *C*-lobe functions exclusively in the recognition of the inhibitory-junction domain [66]. Based on this J-CaM-LD protein model of CPK1, the amino acids of the EF loop region participating in Ca^2+^-binding are shown using the Swiss-PdbViewer (https://spdbv.vital-it.ch/) (Figure 3B). The binding sites of the four EF-hands are D_1_-D_3_-S_5_-E_12_, D_1_-D_3_-S_5_-E_12_, D_1_-D_3_-S_5_-E_12_, and D_1_-D_3_-I_5_-E_12_, respectively (subscripts denote the position of residues corresponding to one single binding loop sequence) (Figure 3B,C).

In in vitro Ca^2+^-binding assay, CPK25 in subgroup I, was found to possess no capability to bind Ca^2+^ while showing constitutive enzymatic activity in a Ca^2+^-independent manner [1,69]. The apparent Ca^2+^-independence correlates with the alterations of its CaM-LD domain. As is shown, the EF1 and EF2 of CPK25 are degenerated while its EF3 and EF4 are lost (Figure 3A,C; Table 2) [1]. In an in vitro kinase activity assay, seven AtCDPKs from subgroups I (CPK2, 4, 5 and 11) and II (CPK3, 9 and 19) were clearly sensitive to calcium with different intensities, whereas six from subgroup III (CPK7, 8, 10, 13, 30 and 32) exhibited low or no calcium sensitivity. Further research was conducted to analyze the sequence degeneration of their Ca^2+^-binding sites, and it was found that five analyzed AtCDPKs (CPK7, 8, 10, 13 and 32) showing lower or no calcium sensitivity all carry one or two altered EF-hand motif(s), suggesting that the degeneration of the EF-hand motifs can greatly influence the calcium loading [1] (Table 2). Similarly, CPK23 with weak calcium sensitivity also possesses a degenerated EF-hand motif [50]. Notably, CPK30, harboring four consensus EF-hand motifs, was shown to possess kinase activity in a calcium-independent manner. It is proposed that some other AtCDPK regions as well as the substrates might also be involved in the kinase stabilization of the inactive or active conformation [1,66].

## 5. Functions of AtCDPKs in the Regulation of Plant Growth and Development

After diverse Ca^2+^ signals are sensed by CDPK and various sensors, the signals are decoded into global signaling events and induce the numerous corresponding responses in plants via diverse targets and pathways involved. The first plant CDPK was reported in *Pisum sativum* in 1984 [70], while AtCDPK study began with *CPK10* and *CPK11* in 1994 [48]. To date, though there are still many perspectives which are largely unclear, longstanding knowledge about CDPKs has been obtained. As for the pivotal roles that CDPKs play in Ca^2+^ signaling, many reviews have been presented, most of which are focused on their subcellular localization, target proteins and functions in plant growth and development, immune and stress signaling, and phytohormone signaling, and the readers can be directed to those presented by Liese et al. [1], Simeunovic et al. [2], and Xu and Huang [71]. Here, we mainly introduce their functions in pollen tube growth, nutrient transport and tolerance regulation to abiotic stresses in plants.

### 5.1. Regulation in Pollen Tube Growth and Elongation

**Figure 4:**
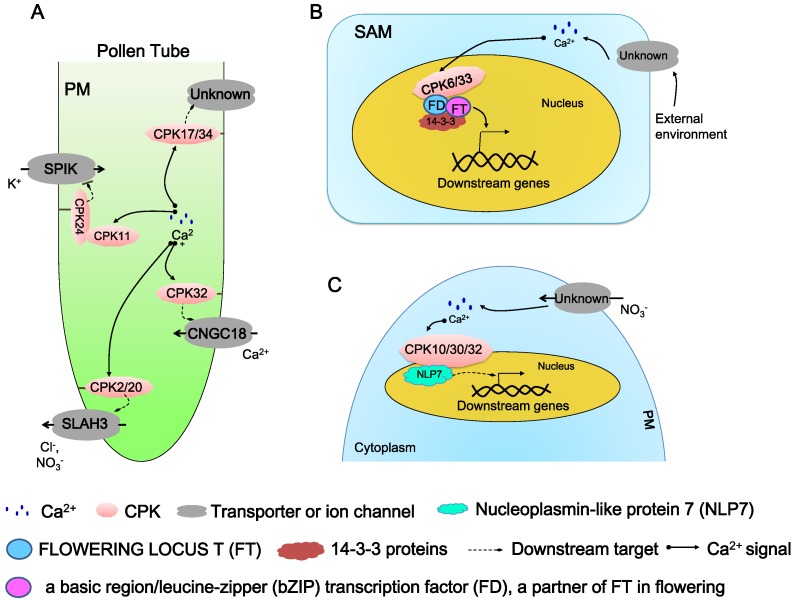
The function of AtCDPKs in the regulation of plant growth and development. The regulation networks of AtCDPKs in regulation of pollen tube elongation (**A**), floral signaling (**B**) and nutrition transport in plant cells (**C**). PM, plasma membrane. SAM, shoot apical meristem. External environment, photoperiod, light quality, and ambient temperature, as well as endogenous cues.

A pollen tube (PT) is a type of specialized plant cell, and its growth exclusively at the tip is a rapid and highly polarized process. There are basically four ions, including Ca^2+^, H^+^, K^+^, and Cl^−^, which participate in the regulation of this process. CDPKs act as an important molecular controller involved in the dynamic ion fluxes and gradients (Figure 4A) [40,62].

Four PT-specific AtCDPKs, CPK2/17/20/34, were verified to regulate the PT growth and elongation. Isoforms CPK17 and CPK34 were found earlier to show a functional redundancy in PT growth and play an important role in PT growth rate and pollen transmission efficiency [53]. Although double mutant *cpk17cpk34* displayed a normal morphology, its PT growth rate and pollen transmission efficiency was seriously impaired compared to wild-type plants [53]. It is supposed that *CPK17* and *CPK34* might be essential to pollen fitness and PT tip growth. The downstream physiological targets need to be further identified [53]. CPK2/CPK20 was identified to influence PT growth by regulating a slow anion channel-associated channel SLAH3 (Figure 4A) [40]. At the beginning of the research, it was noticed that growing pollen tubes possess a cytosolic anion concentration gradient and apical anion oscillations, while the cytosolic Ca^2+^ homeostasis is correlated with anion concentration gradient at the tip. Then, SLAH3 was identified as the only pollen tube anion channel, while four PT-specific AtCDPKs, CPK2/17/20/34, were revealed to localize to the plasma membrane of the PT tip [40]. Among these four proteins, CPK2 and its closely-related homolog CPK20, both from subgroup I, have a specific expression exclusively at the growing PT tips. FRET-FLIM (Förster-resonance energy transfer fluorescence lifetime microscopy) measurement and a BiFC assay were conducted to reveal that there is a specific interaction between CPK2/CPK20 and SLAH3 (Figure 4A) [40]. The two-electrode voltage clamp technique was used to confirm that CPK2 and CPK20 are able to activate SLAH3 and result in instantaneous currents with typical time-dependent S-type anion channel deactivation kinetics [40]. Further anion currents and fluxes in the PT of *slah3-1*, *slah3-2* and *cpk2-1cpk20-2* are reduced [40]. However, the direct interaction of biochemical evidence between CPK2/20 and SLAH3 is still lacking.

Another identified pathway in PT cell is the CPK11-CPK24-SPIK pathway (Figure 4A) [62]. Using patch-clamp analysis, it was found that the K^+^ influx (K^+^_in_) of PT protoplasts was inhibited by elevated cytoplasmic Ca^2+^ concentration. Among 16 PT-expressed CDPKs, mutants for six AtCDPKs differed from wild-type plants in pollen germination or pollen tube growth [62]. From the measurement of K^+^ influx current in *cpk11* and *cpk24* mutants, it was found that both CPK11 and CPK24 are dispensable in the Ca^2+^-dependent inhibition of K^+^_in_ channels, and these two AtCDPKs function in the same pathway [62]. CPK11 is able to bind and phosphorylate CPK24 in vivo. Further electrophysiological experiments indicated that shaker pollen K^+^_in_ channel (SPIK) may act as the target protein in this pathway, which leads us to conclude that CDPK can regulate pollen tube elongation by the K^+^_in_ regulation [62].

The most recent reported pathway in PT is the CPK32-cyclic nucleotide-gated channel (CNGC) 18 pathway (Figure 4A) [55]. Zhou et al. [55,72] found that the respective over-expression of GFP (green fluorescent protein)-tagged CPK14, CPK32 and CPK34 in PT can induce the depolarization of pollen tube growth, and CPK32-GFP over-expression produced the most severe phenotypes, resulting in a short tube with the swelling tip [55]. Ratiometric Ca^2+^ imaging assay indicated that *CPK32* over-expression disrupted the Ca^2+^ homeostasis; cyclic nucleotide-gated channels (CNGCs), a kind of voltage-gated ion channel, were supposed to be involved in this process. CNGC18 was later identified as an interactive partner via yeast two-hybrid assay. CPK32 can activate CNGC18-mediated Ca^2+^ in *Xanopus Oocytes*. Over-expression of *CNGC18* produces a similar phenotype to that triggered by *CPK32* over-expression. A synergistic effect in the depolarization of PT growth was also observed when the two proteins were co-expressed. CPK32-CNGC18 may regulate the PT growth by increasing Ca^2+^ accumulation [55].

### 5.2. Regulation in Floral Signaling

Flowering time is crucial to the sexual reproduction of plants. By sensing the external environment, such as the photoperiod, light quality, and ambient temperature, as well as using endogenous cues, plants select the proper flowering timing [41]. There are complicated regulatory networks explored in *Arabidopsis* and other plants, and the most important component is FLOWERING LOCUS T (FT). FT is a 20 kD protein acting as a mobile floral molecule which is also termed as florigen [41]. It was found that FT forms a complex with its interdependent partner FD, a basic region/leucine-zipper (bZIP) transcription factor, via 14-3-3 proteins [41,73]. It is proposed that threonine phosphorylation at position 282 of FD influences the formation and function of the complex, but that the kinase responsible for this modification was unknown [73]. By in vitro kinase activity assay, three kinases (CPK4/CPK6/CPK33) were identified to efficiently phosphorylate FD T282 in a Ca^2+^ dependent way. Further biochemical, cellular and genetic analyses showed that two AtCDPKs (CPK6 and CPK33) expressed in shoot apical meristem (SAM) directly interact with FD (Figure 4B). A weak but significant late-flowering phenotype was observed in the *cpk33-1* after the alteration of illumination time, indicating CPK33 is more important for the florigen complex formation [41].

### 5.3. Regulation in Nutrient Sensing and Transport

Mineral nutrients are required by all plants. It is very important to understand the process of how plants sense the availability of nutrients, how the signals are decoded and transferred throughout a plant and how the nutrients are transported. Hitherto, only limited AtCDPKs was reported to be correlated with nutrients [74].

The most recently discovered pathway involved in nitrogen sensing is the nitrate-CPK-NLP (NIN-like protein) signaling pathway, identified in *Arabidopsis* (Figure 4C) [58]. A single-cell system to dynamic Ca^2+^ supervision in *Arabidopsis* mesophyll protoplasts was established by employing an ultrasensitive Ca^2+^ biosensor GCaMP6. Under nitrate stimulus, a specific and dynamic Ca^2+^ signature was observed in the nucleus and cytosol of mesophyll protoplasts, and enhanced AtCDPKs kinase activity was detected in response to nitrate within a short time (10 min). To search for AtCDPKs candidates, constitutively active AtCDPKs (AtCPKacs) are conducted and co-expressed with *ProNIR-LUC* (a luciferase reporter gene drove by a nitrate reductase promoter that exhibits a specific physiological nitrate response) in plants [75]. When 0.5 mM KNO_3_ is presented, the protoplasts co-expressing *ProNIR-LUC* with six respective CPKacs (CPK7ac, CPK8ac, CPK10ac, CPK13ac, CPK30ac and CPK32ac) in subgroup III were detected with strong LUC activation, which suggested that nitrate triggers unique Ca^2+^–CDPK signaling. However, single mutants lacked obvious growth phenotypes and influenced the expressional regulation of nitrate-responsive genes, and CPK10 and CPK30 (as well as CPK32 later) were chosen in the further research. A chemical switch (termed as 1-isopropyl-3-(3-methylbenzyl)-1H-pyrazolo [3,4-d] pyrimidin-4-amine or 3MBiP) with the engineered mutant CPK10 (M141G) circumvents embryo lethality and enables conditional analyses of *cpk10cpk30cpk32* triple mutants to define comprehensive nitrate-associated regulatory and developmental programs. CPK10, CPK30 and CPK32 were then found to play a central role in controlling of primary transcription by the RNA sequencing. In an in vivo assay, it was possible to phosphorylate conserved NLP, a key transcription factor of primary nitrate responses with Ca^2+^ dependence [58,75]. More biochemical, cellular and genetic evidence was further obtained to validate this nitrate-CPK-NLP pathway. This suggests that the CPK-NLP signaling relay may be widespread in plants and that the nutrient-coupled Ca^2+^ signaling network integrates transcriptome and cellular metabolism with shoot–root coordination and developmental plasticity in shaping organ biomass and architecture [58].

Ca^2+^ signals have been reported to promote stomatal closure through the inhibition of inward K^+^ channels and the activation of anion channels. The first pioneering progress in K^+^ transport is uncovered in *Vicia faba* guard cells by Luan et al. [76] that some Ca^2+^-dependent phophatases participate in regulating the currents of inward K^+^ channels KAT1 (K^+^ affinity transport 1), a shaker channel subunit natively expressed in *Arabidopsis* guard cells, suggesting that phosphorylation and dephosphorylation, as well as ion transporters of guard cells, play important roles in the regulation of stomatal aperture. A further study indicated that KAT1 is phosphorylated by a 57-kD VfCDPK kinase in a Ca^2+^-dependent manner [77]. Co-expression of KAT1 and a VfCDPK resulted in the inhibition of KAT1 currents in *X. oocytes* [78]. A recent study showed that CPK13, a weak Ca^2+^-dependent protein kinase, inhibits stomatal opening under light-induced conditions and suppresses the current of KAT1 and KAT2 [61]. In an in vitro kinase assay, KAT2 was identified as the phosphorylation target of CPK13, co-localizing to the plasma membrane with CPK13 in plants. These results suggest that *CPK13* may impair stomatal movement by regulating K^+^ flux [61]. The latest research showed that CPK33 also regulates the activity of GORK, the guard cell outward rectifying potassium channel [51].

### 5.4. Regulation in Phytohormone Signaling Pathways

The first evidence for this was that a membrane-localized CDPK was found to be induced in rice by gibberellins (GAs) in 1995 [79]. Since then, many plant CDPKs have been identified to be involved in hormone synthesis or signaling pathways [71]. The integral associations between CDPKs and phytohormones were elaborated by Xu and Huang et al. [71] in this issue, as the readers can refer to. Here, we only briefly touch on those AtCDPKs were involved in this process.

In *Arabidopsis*, CPK28 is identified as a key regulator of gibberellic acid (GA) and jasmonic acid (JA) levels [80,81]. Two independent *cpk28* mutants that showed reduced growth in shoot elongation, disruption in secondary growth and vascular differentiation exclusively upon the transition to the generative phase exhibited an obvious down-regulation in gene transcription of *GA3ox1*, a key enzyme for GA homeostasis, while showing an increased transcription of two NAC transcriptional factors (a kind of transcriptional factors acts key switches for activation of secondary wall biosynthesis; always contains NAM, ATAF1/2, and CUC2 proteins), *NST1* (NAC secondary wall thickening promoting factor1) and *NST3* (secondary wall-associated NAC domain protein1; also called *SND1*) [80]. Phenotypic features of *cpk28* have also been attributed to elevated levels of JA. Further study indicated that *cpk28* mutants displayed an altered balance of JA and GA by up-regulated JA-dependent gene transcription and JA levels during the generative phase [81]. JA biosynthesis or JA signaling is a prerequisite for realization of the *cpk28* growth phenotype, and abolishment of JA biosynthesis or JA signaling led to a full reversion of the *cpk28* growth. CPK28 not only functions in PAMP (pathogen-associatedmolecular pattern)-induced defense signaling in seedlings, but also in JA-dependent developmental processes in stem elongation and vasculature; importantly, independent of JA-mediated stress and defense signaling responses during the adult phase [81].

Some AtCDPKs might also be involved in auxin signaling in plants [82]. CPK3 and CPK4 are able to phosphorylate two patatin-related phospholipase As (*pPLAs*, here refer to PLAIVA and PLAIVB), a kind of enzyme that cleaves phospho- and galactolipids to generate free fatty acids and lysolipids. *PLAIVA* is expressed strongly and exclusively in roots and its null mutants develop reduced lateral root development, suggesting an impaired auxin response. *PLAIVB* is transcriptionally induced by auxin. PLAIVA is phosphorylated strongly by CPK3 and CPK4 while negligibly by CPK6 and CPK29. CPK3 phosphorylates PLAIVA and, to a lesser extent, PLAIVB. As auxin is a major signal of lateral root formation, *pPLAs* in root development with individual roles might associate auxin signaling with AtCDPKs pathway [82].

In ethylene signaling pathways, CPK16 was found to phosphorylate the ethylene biosynthesis enzyme, 1-aminocyclopropane-1-carboxylate (ACC) synthase 7 (ACS7). ACS protein is the key component of the ethylene biosynthesis process, and it functions at the rate-limiting step to convert S-adenosylmethionine (AdoMet) to ACC. Based on the in vitro assay, three CPK16 phosphorylation sites, Ser216, Thr296, and Ser299, were identified, but whether CPK16 functions in plant roots in vivo requires further study [83]. *CPK4* and *CPK11* also play a positive role in ethylene biosynthesis. When a *cpk4cpk11* double mutant was treated by abscisic acid (ABA), its ethylene production was inhibited [84]. CPK4 and CPK11 are able to phosphorylate the *C*–terminus of ACS6 in vitro to enhance the stability of ACS6 [84].

## 6. Function of AtCDPKs in Abiotic Stress Responses and ABA Signaling Transduction

Multiple evidence reveals that AtCDPKs are involved with the response to external stimuli in combination with ABA [2,25,46]. In *Arabidopsis*, numerous experiments have elucidated the roles of CDPKs in various abiotic stresses, such as drought, heat, salinity and cold, etc. [2,25].

**Figure 5:**
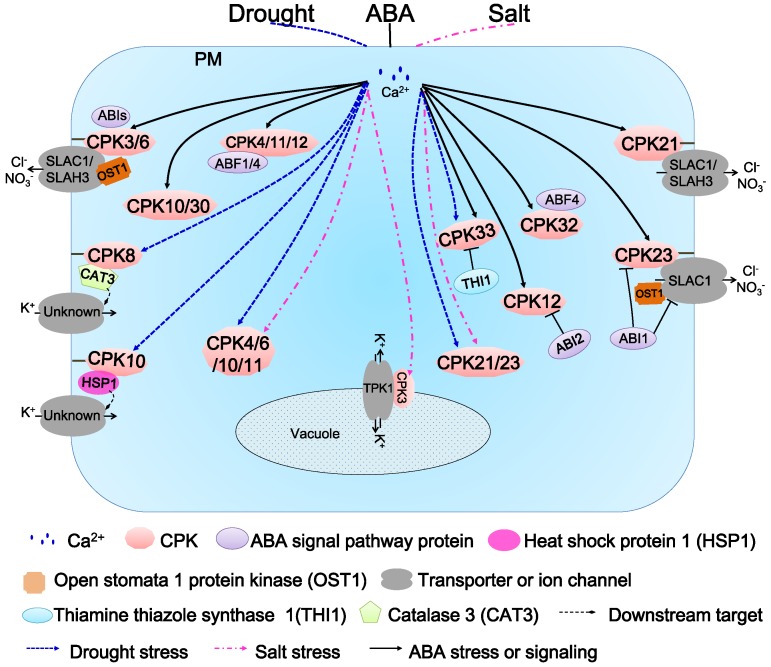
Function of AtCDPKs in abiotic stress responses and abscisic acid (ABA) signaling transduction.

Functional analysis of AtCDPKs began with AtCDPK1 (CPK10) and AtCDPK2 (CPK11). An initial investigation revealed that the *AtCDPK1*(*CPK10*) and *AtCDPK2*(*CPK11*) genes are hastily induced in response to drought and high salinity, while they are not influenced under low temperature and heat stress [48]. The *cpk10* mutant, a T-DNA insertion mutant, recently was found to show a dramatically sensitive phenotype to drought, while over-expressing *CPK10* in *Arabidopsis* enhanced drought tolerance. An impairment of induction of stomatal closure and inhibition of stomatal opening was observed on *cpk10* under 10 μM ABA or 5 mM Ca^2+^, indicating that *CPK10* might play roles in drought and ABA- and Ca^2+^-mediated regulation of stomatal movements (Figure 5) [60]. HSP1, a heat shock protein, was identified as an CPK10-interacting protein using the yeast two-hybrid assay. HSP1 and CPK10 co-localize in plasma membrane [60]. *HSP1* knockout mutant (*hsp1*) plants exhibit similar sensitive phenotypes to those of *cpk10* under drought stress in *Arabidopsis*, suggesting that CPK10 and HSP1 might form a functional complex and play a positive role during the stresses [60].

CPK4, CPK11 and CPK12 are located in the same branch in subgroup I (Figure 1). CPK4 and CPK11 were identified to positively regulate CDPK/calcium-mediated ABA signaling in *Arabidopsis* [46]. Single or double mutant plants of CPK4 and CPK11 displayed pleiotropic ABA insensitive phenotypes and salt insensitivity in seed germination. Over-expressing seedlings of *CPK4* and *CPK11* showed greatly enhanced ABA-sensitivity, whereas only negligible tolerance to salt stress was observed. Two ABA responsive transcription factors, ABF1 and ABF4, are phosphorylated by CPK4 and CPK11 in vitro, suggesting that the two kinases regulate Ca^2+^-dependent ABA signaling through these transcription factors [46]. *CPK12* is ubiquitously expressed in *Arabidopsis* and localizes to both cytosol and nucleus [36]. *CPK12* RNAi lines exhibit ABA hypersensitivity in seed germination and post-germination growth [36,85]. Further studies indicated that CPK12 phosphorylates ABF1 and ABF4 as well as ABA insensitive 2 (ABI2), a type 2C protein phosphatase (PP2C) in vitro (Figure 5), suggesting that CPK12 might be a balancer in ABA signaling pathway [36]. Interestingly, when ABI2 loses all the three putative CDPK phosphorylation sites, the interaction between CPK12 and ABI2 is not affected, indicating that the interaction is a distinct process from phosphorylation event of ABI2 by CPK12 [36].

Another two AtCDPKs analyzed more deeply are CPK3 and CPK6. Early studies of the single or double mutant plants of *CPK3* and *CPK6* showed that the ABA-induced current of the slow-type anion channel was significantly inhibited (Figure 5) [42]. Supplying ABA or Ca^2+^ in the external environment induced a decrease in stomatal aperture of wild-type plants, while that of *cpk3-1cpk6–1* and *cpk3-2cpk6–2* double mutants was barely reduced. Therefore, *CPK3* and *CPK6* may function as positive regulators in the ABA-induced regulation of anion channel in guard cells and enhance ABA- and Ca^2+^-dependent stomatal closure (Figure 5) [42]. *CPK6* is induced by salt/drought stress, and its over-expressing lines enhanced the tolerance to salt/drought stresses [86]. CPK6 was found to weakly interact with slow-sustained anion channel 1 (SLAC1), a guard cell anion channel from *Arabidopsis* in *X. oocytes* [50]. In 2012, Brandt et al. reconstituted a functional ABA signal transduction core from ABA receptors to ion channel activation, including PYR1 (pyrabactin resistance/regulatory component of ABA receptor 1), ABI1, CPK6, and *N*-terminus of SLAC1 in vitro [87]. CPK3 constitutively active in roots and leaves in a strictly Ca^2+^-dependent manner in *planta*, while its activity is induced by high-salt and other stresses in *Arabidopsis* protoplasts [54]. The most recent progress is the identification of the CPK3-TPK1 pathway which enhances salt-stress adaptation (Figure 5) [88]. Vacuole is the main reservoir of various salt ions in plants, providing an ability to resist high salt stress. CPK3 is able to phosphorylate the tandem-pore potassium channel1 (TPK1), localized at the tonoplast, at Ser42 of its *N*-terminus. It was found that CPK3 is constitutively co-expressed with TPK1 and activated by calcium [88].

CPK21 and CPK23 were found to act as negative regulators in *Arabidopsis* responses to hyperosmotic stress and drought/salt stresses, respectively. The individual deficiency of CPK21 and CPK23 enhanced the tolerance to corresponding abiotic stresses, while the over-expressing lines showed reverse phenotypes [49,89]. Biochemical analysis demonstrated that CPK21 and CPK23 are able to phosphorylate the guard cell anion channel SLAC1 in a Ca^2+^-sensitive and Ca^2+^-insensitive (weak Ca^2+^-dependence) manner (Figure 5). As open stomata 1 protein kinase (OST1), ABI1 phosphatase and SLAC1 are identified to be key components of Ca^2+^-independent ABA signaling, a new calcium-dependent ABA signaling pathway in guard cells, composed of CPK21 (strongly dependent)/CPK23 (weakly dependent), SLAC1 and ABI1, was explored [50].

*CPK32* is an ABA signaling component that positively modulates the ABA-responsive gene expression via *ABF4* (Figure 5) [56]. CPK32 interact with ABF4 and phosphorylate ABF4 in vitro. The expression patterns of *CPK32* coincide with those of *ABF4*, induced by high salinity and expressed in most of the vegetative tissues, especially in roots, stigma, anthers, and the abscission zone. Subcellular localization of CPK32 demonstrated that CPK32-GFP was mainly localized in the nucleus and the periphery of the cells, while ABF4 was located in the nucleus. *CPK32* over-expression resulted in enhanced expression of *ABF4*-regulated genes and increased ABA and salt sensitivities during germination at high concentrations of ABA and salt [56].

CPK33 plays a negative role in stomatal closure and slow anion currents [52]. Under drought stress and ABA treatment, two distinct *cpk33* mutants displayed a profoundly smaller stomatal aperture than that of wild type plants and showed drought tolerance and an increased activity of the slow anion channel. *CPK33* over-expressing lines showed the reverse phenotypes to those of mutants, including the impairment of stomatal closure, increased water loss and weaker tolerance to drought. It was found that thiamine thiazole synthase 1 (THI1) physically interacts with CPK33. In an in vitro kinase assay, THI1 repressed CPK33 auto-phosphorylation activity, which is consistent with the phenotypes of the *THI1* over-expressing lines. THI1 might negatively regulate CPK33 activity in ABA-induced guard cell signaling [52].

To date, there has been only one report about the role of *CPK8* in abiotic stresses [57]. Using the kinase domain of CPK8 as a bait, a tetrameric iron porphyrin protein CATALASE3 (CAT3) that catalyzes the dismutation of H_2_O_2_ to water and oxygen was obtained by yeast two-hybrid assay. CPK8 is verified to be able to interact and phosphorylate CAT3 via a series of biochemical methods. Both *cpk8* and *cat3* plants were more sensitive to drought stress than wild-type plants, and ABA and Ca^2+^ inhibition of inward K^+^ currents were diminished in guard cells of those plants. *CPK8* over-expressing lines showed enhanced tolerance to drought stress. It is suggested that CPK8 functions in ABA-mediated stomatal movement in response to drought stress through the regulation of CAT3 [57].

## 7. Perspectives

As CDPKs are evolutionarily conserved from algae to land plants, the Ca^2+^-CDPK signaling network participates in numerous activities in the plants. Diverse Ca^2+^ signatures are generated, then recognized and decoded by specific Ca^2+^sensors, and huge physiological and biochemical responses will be triggered in plants. Although much progress has been made in this field, how complex Ca^2+^ signatures in plant cells are generated to diverse stimuli at the very beginning is still largely unknown. Post-transcriptional modifications of CDPKs in plant cells, including autophosphorylation, myristoylation and palmitoylation, are greatly involved in their subcellular localization and their function. The relationship and the detailed mechanism between these modifications and the localization remain to be further elucidated.

CDPKs have the feature of Ca^2+^-dependence, based upon the contained EF-hands. Although most CDPKs are conserved in amino acid sequences, their EF-hands differ to varying degrees. Some EF-motifs are degenerated or lost, resulting in changes in Ca^2+^ dependence or sensitivities. The Ca^2+^ perception capability of CDPKs might also be influenced by the specific cellular circumstances and the surrounding regulators, as different Ca^2+^ signatures might involve many specific cellular factors. The interactive protein effectors and other surrounding regulators may also participate in Ca^2+^ perception or Ca^2+^ loading of CDPKs.

Despite the fact that tremendous progress in CDPK functional characterization has been made in the past decades by genetic, biochemical and physiological methods, only limited downstream targets were identified, including ion channels, biochemical enzymes and transcriptional factors involved in hormone or abiotic signaling. This might be attributed to the redundancies in CDPK genes hampering the phenotyping, as well as the interaction nature, which is transient, instantaneous and dynamic. Further research is likely to extend the identification and functional characterization of some interactive regulators and discover new phosphorylated substrates involved in the Ca^2+^ perception or signaling transduction in a wider range.

## Figures and Tables

**Table 1:** The concentration of Ca^2+^ in plant organelles.

Organelle	[Ca^2+^]_T_ (mM)	[Ca^2+^]_F_ (nM)	References
Vacuole	80	200~5000	[14,15]
Chloroplast	15	~150	[14]
Apoplast	~1.0	330~500	[20,21]
Endoplasmic reticulum	-	-	[14,15]
Mitochondria	-	~200	[22]
Nucleus	-	~100	[23,24]
Cytoplasm	-	~100	[14]

[Ca^2+^]_T_: total Ca^2+^ concentration; [Ca^2+^]_F_: free Ca^2+^ concentration; -: Not determined.

**Table 2:** Degenerated EF-hand motifs of AtCDPKs.

CDPK	Subgroup	Degenerated EF-Hand	Position of Altered Amino Acidin Ca^2+^-Binding Loop	Ca^2+^-Dependence
CPK25	I	1,2 (3,4 missing)	EF1: G_3_ and EF2: H_3_	No
CPK23	II	1	Q_12_	Weak
CPK7	III	1	R_5_	No
CPK8	III	1	K_3_	Weak
CPK10	III	3	T_6_	Weak
CPK13	III	2,3	EF2: K_3_, K_5_ and EF3: L_9_	Weak
CPK14	III	1	R_5_	Not analyzed
CPK30	III	undetected	undetected	No
CPK32	III	1	R_5_	Weak

The analysis is based on amino acid sequences of the conserved EF-hand motifs and those harbored in AtCDPKs (modified from [1,69]).

## Abbreviations

ABA	Abscisic acid
ABF	ABA responsive transcription factor
ABI	ABA insensitive
ACS6/7	1-aminocyclopropane-1-carboxylate (ACC) synthase 6/7
CAD	CDPK activation domain
CaM	Calmodulin
CAT3	Catalase 3
CBL	Calcineurin B-like protein
CDPK	Calcium-dependent protein kinase
CIPK	Calcineurin B-like interacting protein kinase
CLD	Calmodulin-like domain
CML	Calmodulin-like protein
CNGC	Cyclic nucleotide-gated channel
*C*-EF	*C*-terminal EF-hand pair
EF-hand	Elongation factor hand
ER	Endoplasmic reticulum
FT	FLOWERING LOCUS T
FD	A basic region/leucine-zipper (bZIP) transcription factor
FRET-FLIM	Förster-resonance energy transfer fluorescence lifetime microscopy
GA	Gibberellic acid
GORK	Guard cell outward rectifying potassium channel
HSP1	Heat shock protein1
JA	Jasmonic acid
J-CLD	Junction region-CLD
KAT1	K^+^ affinity transport1
NAC	NAM, ATAF1/2, and CUC2
*N*-EF	*N*-terminal EF-hand pair
NLP7NST1	Nucleoplasmin-like protein7NAC secondary wall thickening promoting factor1
NST3	Secondary wall-associated NAC domain protein1; also called SND1
OST1	Open stomata1 protein kinase
PAMP	Pathogen-associatedmolecular pattern
PM	Plasma membrane
PP2C	Protein phosphatase 2C
SPIK	Shaker pollen K^+^_in_ channel
SAM	Shoot apical meristem
SLAC1	Slow anion channel-associated channel1
SLAH3	Slow anion channel-associated channel1 homolog3
3MBiP	1-isopropyl-3-(3-methylbenzyl)-1H-pyrazolo [3,4-d] pyrimidin-4-amine
THI1	Thiamine thiazole synthase1
TPK1	Tandem-pore potassium channel1
